# On the Use of Raw Vegetables

**Published:** 1810-11

**Authors:** 


### TO DR. LAMBE.

THE respect Mr. Royston feels for Dr. Lambe, cannot be lessened by any difference in opinion. A mis-statement of a fact, even from inadvertence, requires, however, some apology. In the present instance,* if Mr. Royston has wrongly represented Dr. Lambe's directions with regard to the use of *raw vegetable food*, he has been misled by what he deemed a publication of considerable authority. Vid. Ann. Med. Rev. vol. ii. 148. To the great object of Dr. Lambe's investigation, the relieving human nature from *Carcinoma*, the most dreadful of its calamities, the *Medical and Physical Journal* will ever open its pages with alacrity: neither will its EDITORS hold themselves justified in endeavouring to employ wit and sarcasm, for the repression of inquiry. Though they do not feel bound to approve every opinion that may come before them; yet, when the motive is the improvement of a science of undeniable utility, they hope always to be governed by a liberal regard to the feelings of an author; and by an honest desire to give a candid statement of his sentiments.

---

* Med. and Phys. Journal, No. 140, for October, 306.

Violent

